# Assessment of handwashing impact on detection of SARS-CoV-2, *Staphylococcus aureus*, *Escherichia coli* on hands in rural and urban settings of Côte d’Ivoire during COVID-19 pandemic

**DOI:** 10.1186/s12889-024-18838-7

**Published:** 2024-05-22

**Authors:** Sylvain Gnamien Traoré, Gilbert Fokou, Affou Séraphin Wognin, Semone Annick Gertrude Dié, Nogbou Andetchi Aubin Amanzou, Kathrin Heitz-Tokpa, Sopi Mathilde Tetchi, Malik Orou Seko, Aimé Roland Sanhoun, Adjaratou Traoré, Etilé Augustin Anoh, Issaka Tiembre, Marina Koussemon-Camara, Chantal Akoua-Koffi, Bassirou Bonfoh

**Affiliations:** 1 Université Peleforo Gon Coulibaly, Korhogo, Côte d’Ivoire; 2 Centre Suisse de Recherches Scientifiques en Côte d’Ivoire, Abidjan, Côte d’Ivoire; 3 Université Virtuelle de Côte d’Ivoire, Abidjan, Côte d’Ivoire; 4 Institut National d’Hygiène Publique, Abidjan, Côte d’Ivoire; 5 Ecole Inter-Etats des Sciences et Médecine Vétérinaires, Dakar, Sénégal; 6 Université Nangui Abrogoua, Abidjan, Côte d’Ivoire; 7 Centre Hospitalier Universitaire de Bouaké, Bouaké, Côte d’Ivoire; 8 UFR Sciences Médicales de l’Université Alassane Ouattara, Bouaké, Côte d’Ivoire

**Keywords:** Côte d’Ivoire, *Escherichia. coli*, Handwashing, SARS-CoV-2, *Staphylococcus* spp

## Abstract

**Background:**

Handwashing is the first line of hygiene measures and one of the oldest methods of preventing the spread of infectious diseases. Despite its efficacy in the health system, handwashing is often inadequately practiced by populations. This study aimed to assess the presence of SARS-CoV-2, *Escherichia coli* (*E. coli*) and *Staphylococcus aureus (S. aureus)* on hands as indicators of lack of hand hygiene during COVID 19 pandemic.

**Methods:**

A cross-sectional study was conducted in rural Taabo and urban Abidjan (Côte d’Ivoire) from January to September 2021. A total of 384 participants from 384 households were included in the study. The total households were distributed proportionally within various municipalities in the two study areas according to the number of households in each municipality, based on data of the National Institute of Statistics from the 2014 general population census. Hand swabbing of the 384 participants within households (320 in Abidjan and 64 in Taabo) was performed for the enumeration of *E. coli* and *S aureus*, using laboratory standard method and for the detection of SARS-CoV-2 by RT-qPCR. A binary logistic regression model was built with the outcome variable presence of *Staphylococcus* spp. on hands of respondents that was categorized into binary variables, *Staphylococcus* spp. (1 = presence, 0 = absence) for the Risk Ratio estimation. Place of living, sex, handwashing, education and age group were used to adjust the model to observe the effects of these explanatory variables.

**Results:**

No presence of SARS-CoV-2 virus was detected on the hands of respondents in both sites. However, in urban Abidjan, only *Staphylococcus* spp. (Coagulase Negative Staphylococci) was found on the hands of 233 (72.8%, 95%CI: 67.7–77.4) respondents with the average load of 0.56 CFU/ Cm^2^ (95% CI, 0.52–0.60). Meanwhile, in rural Taabo, *Staphylococcus* spp. (Coagulase Negative Staphylococci) and *E. coli* were found on the hands of 40 (62.5%, 95%CI: 50.3–73.3) and 7 (10.9%, 95%CI: 5.4–20.9) respondents with the respective average load of 0.49 CFU/ Cm^2^ (95% CI, 0.39–0.59) and 0.08 CFU/ Cm^2^ (95% CI, 0.03–0.18). Participants living in rural Taabo were less likely to have *Staphylococcus* spp. on their hands (RR = 0.811; 95%IC: 0.661–0.995) compared to those living in urban Abidjan.

**Conclusions:**

No SARS-CoV-2 was detected on the hands of participants in both sites, suggesting that our study did not show direct transmission through hands. No *E. coli* was found in urban Abidjan while *E. coli* was found on the hands of participants in rural Taabo indicating poor hand washing and disinfection practices in rural Taabo. Living in urban Abidjan is statistically associated to having *Staphylococcus* spp. on hands. Further studies are necessary especially to understand to what extent the presence of *Staphylococcus* spp. on hands indicates a higher infection or fecal colonization rates in the case of *E. coli*.

## Background

Hand hygiene is an important measure to prevent disease transmission [1]. Diarrhea is one of the top 10 diseases contributing to global Disability-adjusted life years (DALY) [2]. Furthermore, according to the Global Burden of Diseases Study 2017 reports, there were 3.2 million deaths in the world due to chronic obstructive pulmonary disease and 495,000 deaths due to asthma [3]. Severe acute respiratory syndrome (SARS), Middle East respiratory syndrome (MERS) and COVID-19 (Coronavirus Disease 2019) have emerged in 2002–2003, 2012 and 2019–2020 respectively [4]. Contamination by these viruses and diarrhoea can be prevented through handwashing [5]. A comprehensive analysis of the hand surface components indicated that organic acids, especially lactic acid and antimicrobial peptides, are highly correlated with antimicrobial activity and hand hygiene must be improved to enhance natural antimicrobial activity on the surface of hands [6].

Human hands have been the main carrier for the transmission of infection at home, restaurants and public transport [7]. They are one of the vehicles of the transmission of most infections, including mainly diarrheal and respiratory diseases [8].

Hand hygiene measures have been recommended by health authorities and public health experts worldwide to prevent the spread of SARS-CoV2 via contact with infected people and surfaces [9]. Hand hygiene practices are important not only during the COVID-19 pandemic, they are also critical to prevent the possible spread of other infectious diseases [10]. Moreover, the promotion of handwashing with soap can reduce the risk of diarrhoea by 30% (0·70 [0·64–0·76]) [11] and the risk of acute respiratory infection morbidity by about 17% (RR 0·83 [95% CI 0·76–0·90]) [12]. Proper washing of hands and body reduces or eliminates a large proportion of microorganisms acquired through contact with contaminated surfaces and liquids. Microorganisms are detected everywhere on humans, including the skin, oral cavity, gastrointestinal tract, respiratory tract, and urogenital tract that are colonized by a large variety (10–100 trillion) of microorganisms [13]. Most of the skin microbiota composed of over 100 phylotypes are non-pathogenic [13]. Interactions between bacteria, skin cells and immune cells can repair and reinforce the barrier formed by the skin and the disruption of this interaction can leave the skin susceptible to eczema and skin allergies, or interfere with the healing of people with diabetic ulcers [14]. A causal link exists between hand hygiene and infection transmission [15].

However, many people overlook the importance of handwashing when engaging in activities that require the washing of hands [16]. It is estimated that three out of ten people, i.e., 2.3 billion globally, lack adequate facilities with water and soap to wash their hands at home, including 670 million who have no handwashing facility at all [17]. Handwashing at the household level is determined by several factors, such as knowledge, availability of water, availability of a hand washing facility, number of children under 5 years and availability of soap [18].

In Côte d’Ivoire, hand hygiene was widely recommended by the government as one of the main preventive measures against COVID-19 and Ebola. While most of the population acknowledged the benefit of regular handwashing to prevent disease transmission, many people did not comply with the recommendations [19, 20].

Assessing hand contamination could have a great importance in understanding hygienic practices in Ivorian population. Indicator organisms are frequently used to detect whether contamination is absent or present or within unacceptable limits [21, 22]. Total and fecal coliforms, *Escherichia coli* (*E. coli*), belonging to the Enterobacteriaceae family and *Staphylococcus aureus* are used as indicators to assess hygienic conditions [23]. SARS-CoV-2 viral RNA was found with a prevalence of 40.5% (95% CI: 27.4- 55.1%) in stool of COVID-19 patients [24]. A recent study conducted in Southern Italy shows that SARS-CoV-2 was detected in wastewater and in bivalve mollusks. Nevertheless, it does not only make sense to look into stool but also at presence of virus in the environment and in an alleged mode of transmission (through hands) [25]. In Côte d’Ivoire, to the best of our knowledge, there is a paucity of studies conducted on hand hygiene [26]. Moreover, none has yet been conducted on hand hygiene in households and on the presence of the SARS-CoV-2 virus on hands during the pandemic.

The purpose of the present study was to assess the presence of *E. coli*, *S. aureus* and SARS-CoV-2 on hands in households in urban and rural settings.

## Materials and methods

### Study site

The study was conducted in Côte d’Ivoire in urban Abidjan and rural Taabo, from January to September 2021, a period of high prevalence of COVID-19 pandemic in the country, resulting in 45,560 confirmed cases and 274 deaths, representing a case fatality rate of 0.6% on 17 April 2021 [27]. The urban site of Abidjan, the economic capital city of Côte d’Ivoire, was selected because the first case of COVID-19 was recorded in Abidjan on 11 March 2020, and it has been the epicenter and the main hotspot of pandemic in the country. After Abidjan, the disease gradually spread over the country, affecting all urban and rural health districts. To assess the impact of handwashing on presence of SARS-CoV-2, *Staphylococcus* spp., and *E. coli* on hands in various socioecological and socioeconomic settings, the rural Taabo site located at about 150 km in northwest of Abidjan was also chosen. The selection of Taabo is justified by the fact that it houses a Health and Demographic Surveillance System (HDSS) site, a research platform gathering several communities from different ethnic groups and sociocultural backgrounds [28].

**Fig. 1:**
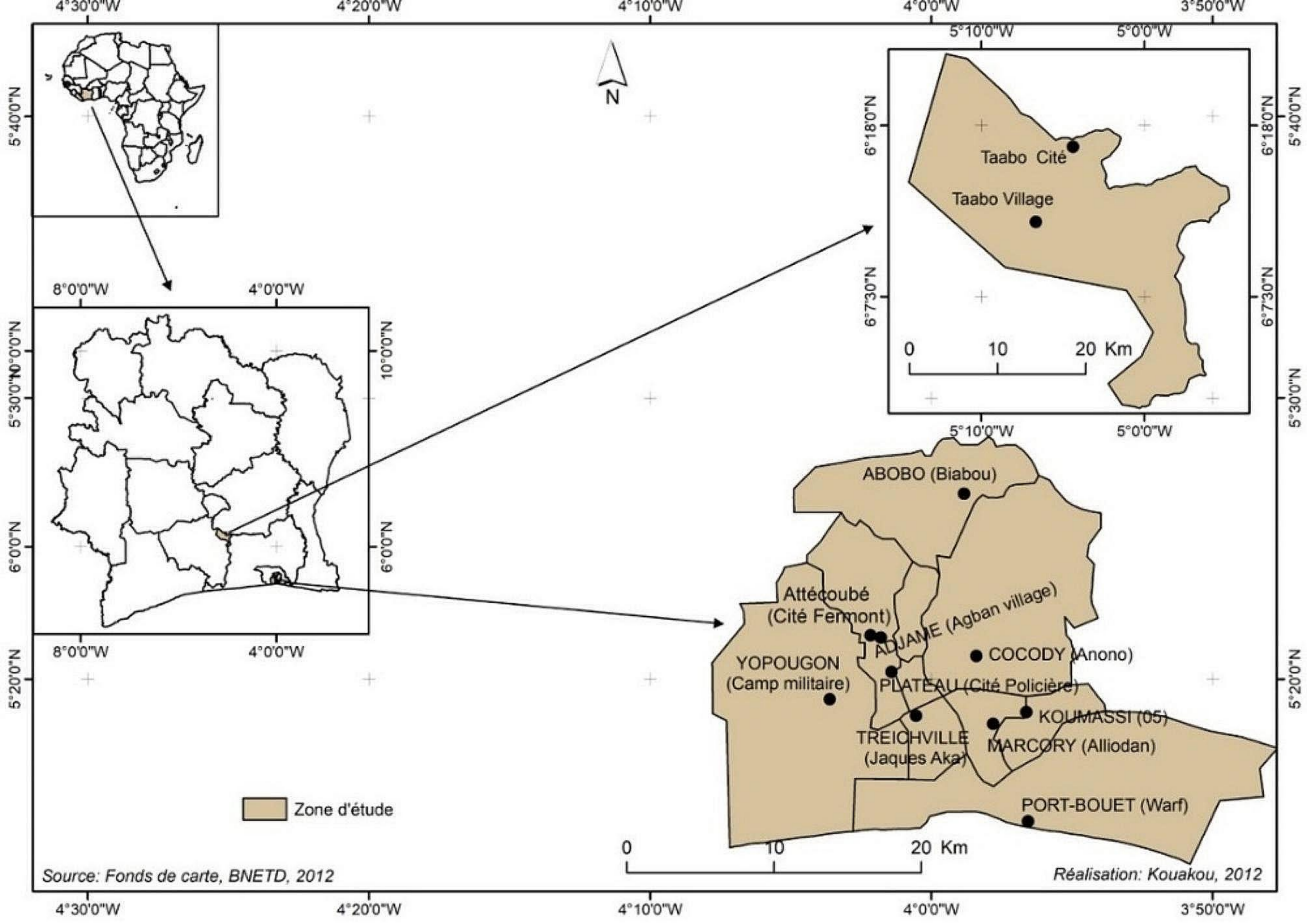
Study sites in Côte d’Ivoire (West Africa)

A municipality is a decentralized geographical and administrative area defined by the local government laws, with a limited autonomy but having powers of self-government. The Abidjan administrative district is subdivided into thirteen (13) municipalities and the study sites in Taabo are located in the rural municipality of Taabo. However, in Abidjan, out of the thirteen municipalities, study sites were selected in ten (Abobo, Adjamé, Attecoubé, Cocody, Koumassi, Marcory, Plateau, Port-Bouët, Treichville and Yopougon). Three peri-urban municipalities (Anyama, Bingerville and Songon) were not included in the study as they are home to semi-rural populations like Taabo. In rural Taabo, the municipality of Taabo was included with two sampling sites (Taabo Cité and Taabo village) selected (Fig. 1). The selection of two sites in Taabo municipality was determined by the demographic and geographical characteristics of the area. The selected sites (Taabo Cité and Taabo Village) are separated by a large artificial lake and come under two different traditional authorities.

### Sample size

The sample size was determined according to the following formula:

$$n= \frac{{1.96}^{2}* {P}_{exp }(1-{P}_{exp})}{{d}^{2}}$$

With *n*, the sample size, *P*_*exp*_, the expected prevalence of presence of bacterial and SARS-CoV-2 on hands in population set at 50% for this study, 95% confidence interval (CI) (Z = 1.96) and *d*, the desired absolute precision (5%) [29].

In total, 384 persons from 384 households were included in the study after obtaining their informed consent.

### Sampling procedure and household’s selection

A total of 384 households were distributed proportionally considering the number of sampling sites in urban Abidjan (ten sampling sites) and rural Taabo (two sampling sites). Thus, the study was conducted in 320 households in urban Abidjan and in 64 households in rural Taabo. The sampling procedure took into consideration the number of households in each municipality in Abidjan and Taabo, based on data of the National Institute of Statistics from the 2014 general population census [30]. The pen method combined with the random number method [31] was used to select households in Taabo and Abidjan. The pen method consists of standing at the central crossroads of the area, throwing a rotating pen in the air to select the investigation direction. The direction on the ground indicated by the head of the pen corresponds to the axis to be investigated. Once a direction is chosen using the “pen” method, the operator takes a table of random numbers and, with his/her eyes closed, randomly points a pen at the table: the number indicated corresponds to the rank of the house from the crossroads where the survey should start. In each household in urban Abidjan and rural Taabo, the household head (or representative) was selected. Visitors or temporary residents (living in the household for less than 6 months) were excluded. In compliance with barrier measures (wearing of face masks and use of hand sanitizer by numerators and researchers with expertise in microbiology), hands of each selected household head (or representative) were swabbed by a researcher with expertise in microbiology, and a structured questionnaire was administered by an enumerator to the selected participant using a mobile computing device (tablet) equipped with Open Data Kit (ODK) software. Discussions during the survey focused on: (i) Socio-demographic characteristics (sex, age group, level of education); (ii) water and soap availability; (iii) handwashing with water and soap before swabbing.

### Sample collection and transportation

A moist swab was used to rub the respondent’s hands. Hand swabbing took place in the morning before 10 AM and was done in duplicate (one for SARS-CoV2 and one for fecal coliforms and *S. aureus*) aseptically using a sterile swab and a tube containing universal transport medium (UTM). Samples were placed in the tube containing the UTM transport medium and sealed. To avoid any possible risk of contamination, samples were transported under secured conditions (triple packaging). Each primary sample was divided into two aliquots in cryotubes, one for the detection of COVID-19 virus and the other for *E. coli* and *S. aureus*. The aliquots for virology were stored in a liquid nitrogen canister and sent to the laboratory of the University Hospital of Bouaké for RT-qPCR on SARS-CoV-2. Those for bacteriological analyses were sent in a cooler containing cold accumulators to the microbiology laboratory of the *Centre Suisse de Recherches Scientifiques en Côte d’Ivoire* in Abidjan.

### Laboratory analysis

#### **Enumeration of *E. coli *and *S. aureus***

The swabs were diluted by adding 100µL of each sample to 900µL of Buffered Peptone Water (BPW). Baird Parker agar (Merck, Darmstadt, Germany) was used for the enumeration of *S. aureus* as described in AFNOR NF 08–057. Inoculation was done by spreading 100 µL of each dilution (10^− 1^ and 10^− 2^) on the surface of the agar previously poured and cooled in Petri dishes. It was incubated at 30 °C for 24 h. Black colonies that were shiny, whole, convex and surrounded by clear areas were considered as *S. aureus* and counted. Petri dishes with bacterial counts between 15 and 150 were considered valid for determining *S. aureus.* Three well-isolated shiny black colonies were selected and tested for confirmation of *S. aureus* (coagulase-positive) a species of Staphylococci with DNAse and coagulase production. As the production of coagulase was negative, the species of Staphylococci are coagulase-negative staphylococci (CoNS). Due to the similar biochemical characteristics exhibited by various coagulase-negative staphylococci (CoNS) species and species heterogeneity [32, 33], we were not able to differentiate phenotypically species. These strains are *Staphylococcus* spp. (Coagulase Negative Staphylococci) others than *S. aureus.*

Rapid E. coli 2 agar culture medium (Bio-Rad, California, United States) was used for the detection and enumeration of *E. coli* according to AFNOR BRD-07/7–12/04(2). Inoculation was done by spreading 100 µL of each dilution (10^− 1^ and 10^− 2^) on the surface of the agar previously poured and cooled in Petri dishes. The incubation was done at 37 °C for 24 h. Pink or purple colonies were identified as *E. coli*. Those with greenish or blue coloration were considered coliforms other than *E. coli*. Plates containing bacterial counts between 15 and 150 colonies were retained to determine *E. coli* loads.

#### Detection of SARS-CoV-2 genes

The detection for the coronavirus was done by the polymerase chain reaction technique called RT q-PCR or Reverse transcription quantitative PCR. We used positive and negative (PCR Water) controls and tested every sample once. Sequences of the primers and probes used in this study came from the study implemented on detection of 2019 novel coronavirus (2019-nCoV) by real-time RT-PCR [34]. The samples were first inactivated in a glove box (PSM3) according to the standard protocols of the Molecular Diagnostic Unit of Hemorrhagic Fevers and Emerging Viruses of the University Hospital of Bouaké, Côte d’Ivoire. One aliquot of the inactivated samples was stored at -80 °C and the second was used for nucleic acid extraction. The first step of the RT q-PCR technique consisted in extracting and purifying the RNA molecules present in the samples to isolate them from the other components. Nucleic acid extraction was performed from 140 µL using the QIAamp viral RNA mini 250 kit (Qiagen, Hilden, Germany) according to the manufacturer’s recommendations. Elution was performed with 140 µL of AVE elution buffer and the final eluate was directly used for PCR. Then an amplification of the RNA sequences present in 3 genes of the virus called E, RdRp, and N, was performed to obtain a sufficient signal for their detection and quantification. As the quantitative PCR technique does not allow the amplification of RNA molecules, reverse transcription (RT) will convert RNA molecules into DNA molecules. PCR was performed using an ABI 7500 Fast cycler (Applied Biosystem Instrument, Germany) by incubating the samples at 94 °C for 10 min, initial denaturation at 94 °C for 3 min followed by 45 cycles consisting of denaturation at 94 °C for 15 s, annealing at 58 °C for 30 s.

The PCR was done first for the detection of the envelope gene (E) with the amplification mixture (Mix) for E gene, then in case of positivity, the confirmation test was done with the Mix for the detection of the RdRP gene. The formation of PCR products was visualized, at the end of each PCR cycle, by incorporating a fluorescent signal to the DNA molecules being formed. The extraction of RNA, the preparation of the “Mastermix” reaction medium and the polymerase chain reaction (PCR) were carried out in different rooms for each step, to avoid possible contamination.

### Data analysis

Data was recorded and analysed using Statistical Package for the Social Sciences (SPSS) version 20 software. The two outcome variables that are presence of *Staphylococcus* spp. and presence of *E. coli* on hands of respondents in rural Taabo and urban Abidjan, were categorized into binary variables, *E. coli* (1 = presence, 0 = absence) and *Staphylococcus* spp. (1 = presence, 0 = absence). Firstly, a Chi-square (χ^2^) or Fischer’s exact test when appropriate was performed to compare prevalence of presence of *E. coli* on hands of respondents and prevalence of presence of *Staphyloccocus* spp. on hands of respondents according to explanatory variables such as sex, site, water availability, education and age group, respectively.

Secondly, the normality distribution of mean of *Staphyloccocus* spp. loads on hands of respondents and mean of *E. coli* loads on hands of respondents were tested with Shapiro-test and showed that data failed normality distribution. Therefore, a Kruskal-wallis test was used when variables are more than 2 categories and Mann–Whitney U test was used when variables are two categories to compare mean of *Staphyloccocus* spp. loads on hands of respondents and mean of *E. coli* loads on hands of respondents, according to previous explanatory variables.

Finally, a binary logistic regression model was built with the outcome variable presence of *Staphylococcus* spp. on hands of respondents in rural Taabo and urban Abidjan, which was categorized into binary variables, *Staphylococcus* spp. (1 = presence, 0 = absence) for the Risk Ratio estimation. Place of living, sex, handwashing, education and age group were used to adjust the model. Interpretation of the significance of the risk ratio was made based on the exclusion of 1 in the 95% confidence interval. A relative risk of one implies there is no difference of the event if the exposure has or has not occurred. If the relative risk is greater than 1, then the event is more likely to occur if there was exposure. If the relative risk is less than 1, then the event is less likely to occur if there was exposure.

## Results

### Socio-demographic characteristics and handwashing

In urban Abidjan, among the 320 respondents, 89 (27.81%) were men and 231 (72.18%) women. In rural Taabo, among the 64 respondents, 27 (42.18%) were men and 37 (57.81%) were women. In the urban site, 142 (44.37) respondents were between 22 and 35 years whereas in the rural site, 15 (23.44%) respondents were between 22 and 35 years (Table 1).

In Abidjan, 86 (26.87%), 66 (20.62%), 60 (18.75%), 28 (8.75%) and 80 (25. 00%) respondents had secondary, primary, university, koranic school levels and no formal education, respectively. In Taabo, however, 27 (42.18) respondents never attended any formal school while 15 (23.44%), 17 (26.56%), and 5 (7.81%) had secondary, primary and koranic school levels, respectively.

Most respondents in rural Taabo, i.e., 60 (93.75%) as well in urban Abidjan, i.e., 280 (87.5%) had tap water in their homes. In rural Taabo, the survey showed that 58 (90.6%) of respondents washed their hands before sampling whereas in Abidjan, 130 (40.6) washed their hands before sampling.

The median time between last handwashing and sampling in urban Abidjan and rural Taabo was 53.5 min (2-209) and 54.5 min (1-211) respectively (Table 1).

**Table 1:** Socio-demographic characteristics of respondents in Abidjan and Taabo households

Characteristics of population samples	Rural Taabo (*n* = 64)	Urban Abidjan (*n* = 320)
	Frequency (%)	Frequency (%)
**Sex**		
Men	27 (42.18)	89 (27.81)
Women	37 (57.81)	231 (72.18)
**Age group**		
≤ 21	2 (3.12)	37 (11.56)
[22 ;35]	15 (23.44)	142 (44.37)
[36 ;45]	15 (23.44)	68 (21.25)
[46 ;55]	16 (25.20)	43 (13.43)
≥ 56	16 (25.20)	30 (9.37)
**Education**		
None	27 (42.18)	80 (25.00)
Koranic school	5 (7.81)	28 (8.75)
Primary	17 (26.56)	66 (20.62)
Secondary	15 (23.44)	86 (26.87)
University	0 (0)	60 (18.75)
**Water availability**		
Tap water at home	60 (93.75)	280 (87.5)
Purchased water	0 (0)	31 (9.69)
Access to pump	4 (6.25)	8 (2.50)
Well	0 (0)	1 (0.31)
**Handwashing prior sampling**		
Yes	58 (90.6)	130 (40.6)
No	6 (9.4)	190 (59.4)
**Time between last handwashing and swabs**		
Median (minutes) (range)	54.5 (1-211)	53.5 (2-209)

No education refers to no attendance to primary, secondary, university and koranic school.

Water availability refers to principal water source for drinking and for any type of use.

The classification of respondents into age groups was determined on the basis of the age of the head of household and according to the following criteria: the age of civil majority (set at 21 in Côte d’Ivoire); the age of the working population (the maximum age for entering the civil service in Côte d’Ivoire with the middle cycle is 35); and the age of the non-working population (the retirement age in Côte d’Ivoire is 57).

### **Prevalence of presence of *E. coli***, ***Staphylococcus *spp. and SARS-CoV-2**

In both rural Taabo and urban Abidjan, among the 384 respondents, no presence of SARS-CoV-2 on hands of the respondents was observed, while *Staphylococcus* spp. was found on the hands of 273 respondents with the prevalence of 71% (95%CI: 66.4–75.4) and *E. coli* was found on the hands of 7 respondents with the prevalence of 2.0% (95%CI: 0.89–3.71) (Table 2). Regarding place of living, in rural Taabo, *Staphylococcus* spp. was found on the hands of 40 respondents with a prevalence of 62.5% (95%CI: 50.3–73.3) and *E. coli* were found on the hands of 7 respondents with the prevalence of 10.9% (95%CI: 5.4–20.9). In urban Abidjan, only *Staphylococcus* spp. was found on the hands of 233 respondents with a prevalence of 72.8% (95%CI: 67.7–77.4). (Table 2).

Regarding to sex, *Staphylococcus* spp. was found on hands of 199 women and 74 men with a prevalence of 74.3% (95%CI: 68.7–79.1) and 63.8% (95%CI: 54.74–71.97), respectively. The difference in the presence of *Staphylococcus* spp. on hands between men and women was statistically significant. However, we did not observe any statistically significant association between the presence of *E. coli* on hands and the sex of participants (Table 2). Neither age group nor water availability were significantly associated with presence of *E. coli* and *Staphylococcus* spp. on hands of respondents (Table 2).

Table 2 shows that 68 respondents with primary and 75 respondents with secondary level education had *Staphylococcus* spp. on their hands with the prevalence of 81.9% (95%CI: 72.3–88.7) and 74.3% (95%CI: 64.9–81.8) respectively. The education level was significantly associated with the presence of *Staphylococcus* spp. on hands of respondents. Regarding handwashing prior to sampling, 119 (63.3%, 95%CI: 56.2–69.9) who washed their hands prior to sampling had *Staphylococcus* spp. on their hands and 7 (3.7%, 95%CI:1.8–7.5) of them had *E. coli* on their hands compared to those who did not wash their hands prior to sampling (0% for *E. coli* and 78.6% for *Staphylococcus* spp.). The presence of *Staphylococcus* spp. and *E. coli* on hands of respondents was statistically associated to handwashing prior to sampling.

**Table 2:** Prevalence of presence of *E. coli*, *Staphylococcus* spp. (Coagulase Negative Staphylococci) and SARS-CoV-2 on the hands of participants within households according to sociodemographic variables

Category	Total	E. coli		Staphylococcus spp.		SARS-CoV-2	
	*N*	*n* (%) (95%CI)	*p*-value	*n* (%) (95%CI)	*p*-value	*n* (%) (95%CI))	*p*-value
**Both sites**	384	7 (2) (0.89–3.71)	NA	273 (71) (66.4–75.4)	NA	0 (0) (NA)	
**Places of living**							
Urban	320	0 (0) (NA)	0.000*	233 (72.8) (67.7–77.4)	0.097	0 (0) (NA)	-
Rural	64	7 (10.9) (5.4–20.9)		40 (62.5) (50.3–73.3)		0 (0) (NA)	
**Sex**							
Women	268	3 (1.1) (0.38–3.24)	0.117	199 (74.3) (68.7–79.1)	0.038*	0 (0) (NA)	
Men	116	4 (3.4) (1.35–8.53)		74 (63.8) (54.74–71.9)		0 (0) (NA)	
**Age group**							
≤ 21	39	0 (0) (NA)	0.674	29 (74.4) (58.9–85.4)	0.264	0 (0) (NA)	
[22 ;35]	157	3 (1.9) (0.6–5.5)		118 (75.2) (67.8–81.3)		0 (0) (NA)	
[36 ;45]	83	1 (1.2) (0.2–6.5)		51 (61.4) (50.7–71.2)		0 (0) (NA)	
[46 ;55]	59	1 (1.7) (0.3-9)		42 (71.2) (58.6–81.2)		0 (0) (NA)	
≥ 56	46	2 (4.3) (1.2–14.5)		33 (71.7) (57.4–82.7)		0 (0) (NA)	
**Education**							
None	107	1 (0.9) (0.2–5.1)	0.457	75 (70.1) (60.8–77.9)	0.016*	0 (0) (NA)	
Koranic school	33	0 (0) (NA)		21 (63.6) (46.6–77.8)		0 (0) (NA)	
Primary	83	3 (3.6) (1.2–10.1)		68 (81.9) (72.3–88.7)		0 (0) (NA)	
Secondary	101	3 (3.0) (1.02–8.4)		75 (74.3) (64.9–81.8)		0 (0) (NA)	
University	60	0 (0) (NA)		34 (56.7) (44.1–68.4)		0 (0) (NA)	
**Water availability**							
Tap water at home	340	7 (2.1) (1-4.2)	0.820	247 (72.6) (67.7–77.1)	0.154	0 (0) (NA)	
Purchased water	31	0 (0) (NA)		19 (61.3) (43.8–76.3)		0 (0) (NA)	
Access to pump	12	0 (0) (NA)		7 (58.3) (31.9–80.7)		0 (0) (NA)	
Well	1	0 (0) (NA)		0 (0)		0 (0) (NA)	
**Handwashing prior sampling**							
Yes	188	7 (3.7) (1.8–7.5)	0.006*	119 (63.3) (56.2–69.9)	0.001*	0 (0) (NA)	
No	196	0 (0) (NA)		154 (78.6) (72.3–83.7)		0 (0) (NA)	

*Statistical significance based on χ 2 or Fisher exact test. NA refers to not applicable.

### ***Staphylococcus *spp. and *E. coli *loads on the hands of participants**

In urban Abidjan, only *Staphylococcus* spp. was found on the hands of respondents with the average load of 0.56 CFU/ Cm^2^ (95%CI: 0.52–0.60). In rural Taabo, *Staphylococcus* spp. and *E. coli* were found on the hands of respondents with the respective average load of 0.49 CFU/ Cm^2^ (95%CI: 0.39–0.59) and 0.08 CFU/Cm^2^ (95%CI: 0.03–0.18). No statistically significant difference of *Staphylococcus* spp. loads on hands of respondents was observed between places of living (Table 3).

*Staphylococcus* spp. was observed on the hands of women and men within households with the average load of 0.58 CFU/ Cm^2^ (95%CI: 0.54–0.63) and 0.48 CFU/ Cm^2^ (95%CI: 0.41–0.56), respectively. No statistically significant difference of *Staphylococcus* spp. loads on hands of respondents was observed between sex. We did not also observe any statistically significant difference of *E. coli* and *Staphylococcus* spp. loads on hands of respondents between age groups as well as the types of available water (Table 3).

Table 3 shows that the load of *Staphylococcus* spp. on hands of respondents was significantly associated with their education level and the handwashing before sampling. The loads of *Staphylococcus* spp. on the hands of participants with primary education level (0.65 CFU/ Cm^2^, 95%CI: 0.58–0.73) were higher than those with koranic school level (0.48 UFC/ Cm^2^; 95%CI: 0.33–0.62), and those with university education level (0.42 CFU/ Cm^2^, 95%CI: 0.32–0.53), who had the lowest loads. Participants who washed their hands before sampling (0.49 CFU/ Cm^2^, 95%CI: 0.43–0.55) had the lower average load of *Staphylococcus* spp. compared to those who did not wash their hands (0.61 CFU/ Cm^2^, 95%CI: 0.56–0.66).

**Table 3:** Microbial load (CFU/ Cm^2^) on hands of respondents within households according to sociodemographic variables

Indicator organisms	E. coli	*p* value	Staphylococcus spp.	*p* value	SARS-CoV-2
Places of living					
Urban	0 ^a^	0.000*	0.56 (0.52–0.60)	0.100	0
Rural	0.08 (0.03–0.18) ^b^		0.49 (0.39–0.59)		0
Sex					
Women	0.01(0-0.03) ^a^	0.117	0.58 (0.54–0.63) ^a^	0.009	0
Men	0.03 (0.01–0.08) ^a^		0.48 (0.41–0.56) ^a^		0
Age group					
≤ 21	0	0.644	0.59 (0.47–0.71)	0.770	0
[22 ;35]	0.01 (0-0.05)		0.57 (0.52–0.63)		0
[36 ;45]	0.01 (0-0.07)		0.49 (0.39–0.58)		0
[46 ;55]	0.02 (0-0.09)		0.56 (0.46–0.67)		0
≥ 56	0.02 (0-0.12)		0.55 (0.44–0.66)		0
Education					
None	0.009 (0-0.052)	0.351	0.56 (0.48–0.64) ^ab^	0.008*	0
Koranic school	0		0.48 (0.33–0.62) ^a^		0
Primary	0.02 (0-0.09)		0.65 (0.58–0.73) ^b^		0
Secondary	0.02 (0-0.07)		0.57 (0.49–0.64) ^ab^		0
University	0		0.42 (0.32–0.53) ^a^		0
Water availability					
Tap water at home	0.01 (0-0.03)	0.821	0.56 (0.52–0.60)	0.313	0
Purchased water	0		0.49 (0.33–0.64)		0
Access to pump	0		0.45 (0.18–0.67)		0
Well	0		0		0
Handwashing prior sampling					
Yes	0.02 (0.01–0.05) ^a^	0.006*	0.49 (0.43–0.55) ^a^	0.006*	0
No	0 ^b^		0.61 (0.56–0.66) ^b^		0

Variables are expressed as mean (95% confidence interval); For each variable, similar letters in the same column indicate no significant statistical difference and different letters indicate a significant statistical difference (Kruskal-wallis test or Mann–Whitney U test for two categories) when appropriate.

### **Risk factor of presence of *Staphylococcus *spp. on hands of participants to the survey in Taabo and Abidjan**

Table 4 shows that place of living is the factor that significantly affects the presence of *Staphylococcus* spp. on the hands of participants. Participants living in rural Taabo were less likely to have *Staphylococcus* spp. on their hands (RR = 0.811; 95%IC: 0.661–0.995) compared to those living in urban Abidjan.

**Table 4:** Risk factor of presence of *Staphyloccocus* spp. on hands of respondents

Place of living	RR (95% CI)
Rural	0.811 (0.661–0.995)*
**Handwashing**	
Yes	0.886 (0.769–1.021)
**Education**	
Koranic school	0.883 (0.66–1.18)
Primary	1.182 (1.00-1.39)
Secondary	1.026 (0.857–1.228)
University	0.788 (0.602–1.032)
**Sex**	
Men	0.922 (0.783–1.086)
**Age group**	
[22 ;35]	0.989 (0.807–1.211)
[36 ;45]	0.808 (0.627–1.042)
[46 ;55]	0.96 (0.752–1.225)
≥ 56	0.98 (0.76–1.264)

Significance of the RR (risk ratio) was made based on the exclusion of 1 in the 95% confidence interval. References are women for sex, urban for place of living, ≤ 21 for age group, no for handwashing and no formal education for education.

## Discussion

The purpose of the study was to contribute to estimate the prevalence of the presence of *S. aureus*, *E. coli* and SARS-CoV-2 on human hands among general population. This was the first study to focus on presence of *S. aureus*, *E. coli* and SARS-CoV-2 on human hands in Côte d’Ivoire using a community-based survey.

In the investigated households in Abidjan (urban) and Taabo (rural), no presence of SARS-CoV-2 on human hands had been observed. The SARS-CoV-2 can be recovered from non-porous surfaces for at least 28 days at ambient temperature and relative humidity (20 °C and 50% RH). Moreover, increasing the temperature while maintaining humidity drastically reduced the survivability of the virus to as little as 24 h at 40 °C in environment [35]. The absence of SARS-CoV-2 on the hands of all respondents in Taabo and Abidjan could be explained by the fact that all the infected individuals were hospitalised or isolated. The second explanation could be related to the fact that the participants involved in the study were relatively healthy. This second explanation is supported by the study implemented on SARS-CoV-2 contamination on healthy individuals’ hands in community settings during the COVID-19 pandemic in Japan [36]. Findings from our study are in line with those of this study which shows that the detection rate of SARS-CoV-2 RNA from the hands of healthy individuals was extremely low, and no viable viruses were detected on their hands. Findings from our study are different to those from other studies. A study conducted in Cleveland (Ohio State, USA), between November 2020, and April 2021, with symptomatic COVID-19 patients showed that hand samples were positive for RNA of SARS-CoV-2 among 75% of selected patients [37]. Another study conducted between April 2020 and March 2021 in Canada hospitals on 75 hospitalized COVID-19 patients showed infectious SARS-CoV-2 (6 × 10^1^ to 2.3 × 10^2^ Plaque-Forming Units/mL) (PFU/mL) in hand swab samples [38]. The two above mentioned studies were conducted in a hospital context on COVID-19 patients while our study was conducted in a community context with individuals that did not present any symptom of COVID-19.

Concerning the assessment of bacteriological quality of handwashing within households, no presence of *E. coli* on hands of respondents in urban Abidjan was observed but *E. coli* was found on hands of participants within households of rural Taabo with the prevalence of 10.9%. The presence of *E. coli* on the hands of household members in rural Taabo could be explained by a recent fecal contamination [39] and poor hand washing and disinfection practices. *E. coli* have been identified in developing countries as responsible for diarrhea and used as an indicator of fecal contamination [40].

The difference of presence of *E. coli* on hands of respondents in Abidjan and Taabo could be explained by the fact that water, sanitation and hygiene (WASH) coverage levels are higher in urban Abidjan than in rural Taabo [41]. This finding could be also explained by the fact that open defecation, inadequate sanitation and hygiene behavior were common in Taabo [42, 43].

This finding on the absence of *E. coli* detection on the hands of all respondents in urban Abidjan compared to rural Taabo is different from the conclusions of a systematic review conducted on prevalence and concentrations of fecal indicator microorganisms (i.e., *E. coli*, fecal coliform) and enteric pathogens on hands of people in community or household settings. In 84 studies identified, the most common indicators were *E. coli* (56 studies) and there was no significant difference in *E. coli* prevalence between urban and rural areas [41].

The presence of *E. coli* was not related to sex, the age group of respondents or water availability. This result indicates that men as well as women, but also young adults, adults and the elderly practiced inadequate handwashing even when water was available [44].

In urban Abidjan, only *Staphylococcus* spp. (Coagulase Negative Staphylococci) was found on the hands of respondents with the prevalence of 72.8% whereas in rural Taabo, the prevalence of the same bacteria on the hands of respondents was 62.5%. The isolation of *Staphylococcus* spp. (Coagulase Negative Staphylococci) on the hands of respondents in households could be due to the fact that *Staphylococcus* spp. (Coagulase Negative Staphylococci) is present on the skin as commensal because *Staphylococci* are often found in the human nasal cavity (and on other mucous membranes) as well as on the skin [33]. However, this opportunistic pathogen bacterium, can escape our immune defenses, hide for prolonged periods of time asymptomatically in human body, even within blood cells, leading to an immune imbalance and disease development [45]. A flora of the skin, *Staphylococcus* spp., is associated with toxic shock syndrome, boils, impetigo, cellulitis and food poisoning [46], urinary tract infections, prostatitis, acute pyelonephritis and epididymitis [47]. Our findings are in line with those obtained in other studies reporting that the most commonly found bacterial isolate on hands was *Staphylococcus* spp. (Coagulase Negative Staphylococci) as a member of normal skin flora [48, 49].

Regarding the bacterial loads on the hands of participants, in rural Taabo, *E. coli* was found with the average load of 0.08 CFU/ Cm^2^ (95%CI: 0.03–0.18). This finding could be explained by the fact that in areas where human and animal excreta are disposed in an unhygienic way, hands are more contaminated. It is typically the case of rural area where animal husbandry within the household environment is common and exposure to human and animal faeces is of particular concern [50].

The loads of *Staphylococcus* spp. on the hands of participants with primary education level (0.65 CFU/ Cm^2^, 95%CI: 0.58–0.73) were higher than on the hands of those with secondary school level (0.57 CFU/ Cm^2^/, 95%CI: 0.49–0.64) and university education level (0.42 CFU/ Cm^2^, 95%CI: 0.32–0.53), who had the lowest loads. This result suggests that people with a university education have more knowledge of hygiene and are aware of the importance of its application for their health and well-being. This finding is in line with the conclusions of a study conducted on the general quality of handwashing and hand-hygiene practices of the population of Hong Kong. In that study, the authors showed that participants with higher educational levels had fewer missed areas of the hands when washing and they performed handwashing on a more regular basis [51]. People who are more educated are more aware of risks associated with poor hygiene and may adopt healthier lifestyles for their health and well-being [52].

Binary logistic regression analysis also revealed that participants living in rural Taabo are less risky (RR = 0.811; 95%IC 0.661–0.995) to have *Staphylococcus* spp. on their hands compared to those living in urban Abidjan. This finding could be explained by the differences in the skin microbiome of rural and urban residents due to environmental factors. This may be associated with a different degree of exposure to microorganisms from the soil, water, and biomass used in agriculture or livestock [53]. Moreover, in the rural site, human–animal interactions can change the skin microbiome composition resulting in the decline in *Staphylococcus* numbers [54]. The reduction of the number of *Staphylococcus* spp. due to human–animal interactions in rural areas could explain the fact that participants living in rural Taabo are less risky to have *Staphylococcus* spp. on their hands compared to those from urban Abidjan.

This study is among the very few studies using hand swab sampling to estimate the prevalence of *S. aureus* and *E. coli* among population. Several recent studies have assessed *S. aureus* and *E. coli* colonization among hospitalized and non-hospitalized persons. Many among them focused on special populations and specific age groups, such as food handlers [26, 55, 56], healthcare workers [57], children [58] and elderly persons [59]. Most of them used nasal swabbing to estimate prevalence of *S. aureus* [59–61].

Our study has several limitations. The first one is inherent to the cross-sectional design, precluding differentiation between persistent and intermittent carriage of *E. coli* and *Staphylococcus* spp. on hands of participants. Secondly, the swabbing was conducted only on hands of respondents and was not coupled with nasal sampling. Therefore, we might have underestimated the colonization prevalence of *S. aureus* and SARS-CoV-2. The third limitation is that in the households, only one person (the household heads or representative) was swabbed.

Despite these shortcomings, our study sheds new light on the estimation of prevalence of the presence of *E. coli*, *Staphylococcus* spp. and SARS-CoV-2 on hands in the households of urban Abidjan and rural Taabo.

## Conclusions

We present one of the first studies on hand hygiene in households’ impact on detection of the SARS-CoV-2 virus and two selected hygiene indicators on hands in Côte d’Ivoire. No SARS-CoV-2 virus was detected, showing probably low contribution of the hands in the virus dissemination in a period of pandemic. No *E. coli* was found in urban Abidjan while *E. coli* is found on the hands of participants in rural Taabo indicating poor hand washing and disinfection practices in rural Taabo. Living in urban Abidjan is statistically associated to having *Staphylococcus* spp. on hands. Further studies are necessary especially to understand to what extent the presence of *Staphylococcus* spp. on hands indicates a higher infection or fecal colonization rates in the case of *E. coli*.

## Data Availability

The dataset with the manuscript is available for open access to reviewers and other researchers during the review processes.

## Abbreviations

AFNOR: Association française de normalization [French national organization for standardization
BPW: Buffered Peptone Water
CFU: colony-forming unit
CI: Confidence interval
COVID-19: Coronavirus Disease 2019
DALY: Disability-adjusted life years
DNA: Deoxyribonucleic acid
*E. coli*: *Escherichia coli*
GHO: Global Health Observatory
HDSS: Health and Demographic Surveillance System
MERS: Middle East respiratory syndrome
ODK: Open Data Kit
PCR: Polymerase chain reaction
PFU: Plaque-Forming Units
RH: Relative humidity
RNA: Ribonucleic acid
RR: Risk Ratio
SARS: Severe acute respiratory syndrome
SD: Standard deviation
UNICEF: United Nations of International Children’s Emergency Fund
UTM: Universal transport medium
WASH: water, sanitation and hygiene
WHO: World Health Organization
χ^2^: Chi-square tests

## References

[1] MacLeod C, Braun L, Caruso BA, Chase C, Chidziwisano K, Chipungu J (2023). Recommendations for hand hygiene in community settings: a scoping review of current international guidelines. BMJ Open.

[2] Behera DK, Mishra S (2022). The burden of diarrhea, etiologies, and risk factors in India from 1990 to 2019: evidence from the global burden of disease study. BMC Public Health.

[3] Giovanni V, Sara M, Salvatore F (2020). Sandra B Global Burd Chronic Respiratory Dis.

[4] Shi Z, From SARS (2021). MERS to COVID-19: a journey to understand bat coronaviruses. Bull Acad Natl Med.

[5] Luigi C, Fulvio P, Caterina L, Venerando R, Daniela M, Raluca EM (2020). COVID-19 pandemic: Prevention and protection measures to be adopted at the workplace. Sustainability.

[6] Nishioka Y, Nagano K, Koga Y, Okada Y, Mori I, Hayase A (2021). Lactic acid as a major contributor to hand surface infection barrier and its association with morbidity to infectious disease. Sci Rep.

[7] Birteksöz TAS, Erdoğdu G (2017). Microbiological burden of public transport vehicles. Istanbul J Pharm.

[8] WHO.Shaping the. future.The world health report 2003:193 P.

[9] WHO, World Health Organisation (2020). Coronavirus disease 2019 (COVID-19). Situation Rep.

[10] Dwipayanti NMU, Lubis DS, Harjana NPA (2021). Public Perception and Hand Hygiene Behavior during COVID-19 pandemic in Indonesia. Front Public Health.

[11] Wolf J, Hubbard S, Brauer M, Ambelu A, Arnold BF, Bain R (2022). Effectiveness of interventions to improve drinking water, sanitation, and handwashing with soap on risk of diarrhoeal disease in children in low-income and middle-income settings: a systematic review and meta-analysis. Lancet.

[12] Ross I, Bick S, Ayieko P, Dreibelbis R, Wolf J, Freeman MC (2023). Effectiveness of handwashing with soap for preventing acute respiratory infections in low-income and middleincome countries: a systematic review and meta-analysis. Lancet.

[13] Goraya MU, Li R, Mannan A, Gu L, Deng H, Wang G (2022). Human circulating bacteria and dysbiosis in non-infectious diseases. Front Cell Infect Microbiol.

[14] Eisenstein M (2020). The skin microbiome. Nature.

[15] Gizaw Z, Yalew AW, Bitew BD, Lee J, Bisesi M (2022). Effects of local handwashing agents on microbial contamination of the hands in a rural setting. BMJ Open.

[16] Omari R, Zotor F, Baah-Tuahene S, Arthur W (2022). Handwashing knowledge, attitudes, and practices in Ghana. J Prev Med Hyg.

[17] WHO., UNICEF.United Nations Children’s Fund and World Health Organization. State of the World’s Hand Hygiene: a global call to action to make hand hygiene a priority in policy and practice. WHO 2021:87 P.

[18] Abebe A, Debela BG, Sisay W, Assefa Zenebe D, Endashaw Hareru G, Ashuro H (2023). Mothers’ hand washing practices and associated factors among model and non-model households in the rural community of Bibugn district, north west Ethiopia: the context of the Ethiopian health extension package. Heliyon.

[19] Yapi RB, Houngbedji CA, N’Guessan DKG, Dindé AO, Sanhoun AR, Amin A (2021). Knowledge, attitudes, and practices (KAP) regarding the COVID-19 outbreak in Côte d’Ivoire: understanding the non-compliance of populations with non-pharmaceutical interventions. Int J Environ Res Public Health.

[20] Traoré SG, Fokou G, Wognin AS, Heitz-Tokpa K, Tetchi SM, Ouattara FA (2023). Preventive measures against Ebola and COVID-19 and their impact on human dirty-hand Disease Mitigation in Côte d’Ivoire. Sustainability.

[21] Manju G, Santosh KM (2021). Microbiological environmental monitoring in food processing. Indian Food Ind Mag.

[22] Traoré SG, Ndour APN, Ossebi W, Seko MO, Fokou G, Alonso S (2021). Impact of good hygiene management practices on the reduction in microbial contamination of roasted sheep meat sold at urban dibiteries in Senegal. Food Prot Trends.

[23] Alves A, Viveiros C, Lopes J, Nogueira A, Pires B, Afonso AF (2021). Microbiological Contamination in Different Food Service Units Associated with Food Handling. Appl Sci.

[24] Parasa S, Desai M, Thoguluva Chandrasekar V, Patel HK, Kennedy KF, Roesch T (2020). Prevalence of gastrointestinal symptoms and fecal viral shedding in patients with Coronavirus Disease 2019: a systematic review and Meta-analysis. JAMA Netw Open.

[25] Lombardi A, Voli A, Mancusi A, Girardi S, Proroga YTR, Pierri B (2023). SARS-CoV-2 RNA in Wastewater and Bivalve Mollusk samples of Campania, Southern Italy. Viruses.

[26] Kouame KA, Bouatenin KMJP, Coulibaly WH, Djue YF, Dje KM (2020). Evaluation des connaissances, des attitudes et des pratiques en matiere d’hygiene et de securite alimentaire des vendeurs de la viande de poulets braisee en Cote d’Ivoire. Agronomie Africaine.

[27] Milleliri JM, Coulibaly D, Lamontagne F. [Covid-19 in Côte d’Ivoire (March 2020 - April 2021) a year under the seal of the Coronavirus].Medecine tropicale et sante internationale. 2021;1(2). 10.48327/MTSIMAGAZINE.N1.2021.102 PMC9137277 35685041

[28] Koné S, Baikoro N, N’Guessan Y, Jaeger FN, Silué KD, Fürst T (2015). Health & Demographic Surveillance System Profile: the Taabo Health and demographic Surveillance System. Côte d’Ivoire Int J Epidemiol.

[29] Thrusfield M. Veterinary epidemiology.3th ed Oxford: Blackwell Science. 2007:610 p.

[30] INS.RGPH-2014 Résultats globaux. 2014:12 P.

[31] Grais FR, Rose AMC, Guthmann JP. Don’t spin the pen: two alternative methods for second-stage sampling in urban cluster surveys. Emerg Themes Epidemiol. 2007; 4(8). 10.1186/1742-7622-4-8 PMC1894792 17543102

[32] Asante J, Amoako DG, Abia ALK, Somboro AM, Govinden U, Bester LA, Essack SY (2020). Review of clinically and epidemiologically relevant Coagulase-negative Staphylococci in Africa. Microb Drug Resist.

[33] Becker K, Heilmann C, Peters G (2014). Coagulase-negative staphylococci. Clin Microbiol Rev.

[34] Corman VM, Landt O, Kaiser M, Molenkamp R, Meijer A, Chu DK (2020). Detection of 2019 novel coronavirus (2019-nCoV) by real-time RT-PCR. Euro Surveill.

[35] Riddell S, Goldie S, Hill A, Eagles D, Drew TW (2020). The effect of temperature on persistence of SARS-CoV-2 on common surfaces. Virol J.

[36] Matsui H, Sugamata M, Endo H, Suzuki Y, Takarabe Y, Yamaguchi Y (2024). SARS-CoV-2 contamination on healthy individuals’ hands in Community Settings during the COVID-19 pandemic. Cureus.

[37] Redmond SN, Li DF, Haq MF, Jones LD, Nguyen AM, Tiktin M, et al. Frequent detection of severe acute respiratory syndrome coronavirus 2 (SARS-CoV-2) RNA on hands and skin of patients with coronavirus disease 2019 (COVID-19). Infect Control Hosp Epidemiol. 2022;43(12):1976–7. 10.1017/ice.2021.403 PMC8446585 34486504

[38] Lin Y-C, Malott RJ, Ward L, Kiplagat L, Pabbaraju K, Gill K (2022). Detection and quantification of infectious severe acute respiratory coronavirus-2 in diverse clinical and environmental samples. Sci Rep.

[39] da Silva DTG, Ebdon J, Okotto-Okotto J, Ade F, Oscar Mito O, Wanza P (2020). A longitudinal study of the association between domestic contact with livestock and contamination of household point-of-use stored drinking water in rural Siaya County (Kenya). Int J Hyg Environ Health.

[40] Navab-Daneshmand T, Friedrich MND, Gächter M, Montealegre MC, Mlambo LS, Nhiwatiwa T (2018). *Escherichia coli* Contamination across multiple environmental compartments (soil, hands, drinking Water, and Handwashing Water) in Urban Harare: correlations and risk factors. Am J Trop Med Hyg.

[41] Cantrell ME, Sylvestre É, Wharton HC, Scheidegger R, Curchod L, Gute DM (2023). Hands are frequently contaminated with fecal Bacteria and enteric pathogens globally: a systematic review and Meta-analysis. ACS Environ Au.

[42] Hürlimann E, Silué KD, Zouzou F, Ouattara M, Schmidlin T, Yapi RB (2018). Effect of an integrated intervention package of preventive chemotherapy, community-led total sanitation and health education on the prevalence of helminth and intestinal protozoa infections in Côte d’Ivoire. Parasites Vectors.

[43] Diarrasouba W. Analyse situationnelle de l’accès à l’eau, l’hygiène et l’assainissement dans les Ecoles primaires de Taabo, Sud de la Côte d’Ivoire. Mémoire présenté pour l’obtention du Diplôme de Master en Sciences et Gestion de l’Environnement Option: Géosciences et Environnement Université Nangui Abrogoua. 2021:52 P.

[44] Lawson A, Cameron R, Vaganay-Miller M. An evaluation of the Hand Hygiene Behaviour and Compliance of the General Public When Using Public Restrooms in Northern Ireland (NI) during the initial weeks of the Novel Coronavirus (COVID-19) pandemic. Int J Environ Res Public Health. 2021; 18(12). 10.3390/ijerph18126385 PMC8296206 34204779

[45] Raineri EJM, Altulea D, van Dijl JM. Staphylococcal trafficking and infection-from ‘nose to gut’ and back. FEMS Microbiol Rev. 2022; 46(1). 10.1093/femsre/fuab041 PMC8767451 34259843

[46] Sigudu TT, Oguttu JW, Qekwana DN (2023). Prevalence of *Staphylococcus* spp. from human specimens submitted to diagnostic laboratories in South Africa, 2012–2017. S Afr J Infect Dis.

[47] Djawadi B, Heidari N, Mohseni MUTI. Caused by *Staphylococcus saprophyticus*. Urinary Tract Infections - New Insights IntechOpen; 2023.

[48] Zefenkey Z (2022). The impact of the Three Most Common Hand Celansing methods on the Bacterial Profile: a Randomized Clinical Trial. Iberoam J Med.

[49] Yılmaz ES, Çetin SK. Investigation of *Staphylococcus* spp. and *Escherichia coli* Colonization and Biofilm Formation on University Students’ Mobile Phones and Hands. Celal Bayar University Journal of Science. 2017; 13(4): 839 – 44.

[50] Gizaw Z, Yalew AW, Bitew BD, Lee J, Bisesi M (2022). Effects of local handwashing agents on microbial contamination of the hands in a rural setting in Northwest Ethiopia: a cluster randomised controlled trial. BMJ Open.

[51] Wong JSW, Lee JKF. The Common Missed Handwashing Instances and Areas after 15 Years of Hand-Hygiene Education. Journal of Environmental and Public Health. 2019; 2019:5928924. 10.1155/2019/5928924 PMC6702815 31485238

[52] Cerf ME (2023). The social-education-economy-health nexus, development and sustainability: perspectives from low- and middle-income and African countries. Discover Sustain.

[53] Prescott SL, Larcombe DL, Logan AC, West C, Burks W, Caraballo L (2017). The skin microbiome: impact of modern environments on skin ecology, barrier integrity, and systemic immune programming. World Allergy Organ J.

[54] Skowron K, Bauza-Kaszewska J, Kraszewska Z, Wiktorczyk-Kapischke N, Grudlewska-Buda K, Kwiecińska-Piróg J et al. Human skin microbiome: impact of intrinsic and extrinsic factors on skin microbiota. 2021; 9(3):543. 10.3390/microorganisms9030543 PMC7998121 33808031

[55] Woh PY, Thong KL, Lim YAL, Behnke JM, Lewis JW, Mohd Zain SN (2017). Microorganisms as an Indicator of Hygiene Status among migrant food handlers in Peninsular Malaysia. Asia Pac J Public Health.

[56] Beyene G, Mamo G, Kassa T, Tasew G, Mereta ST (2019). Nasal and Hand Carriage Rate of *Staphylococcus aureus* among Food handlers Working in Jimma Town, Southwest Ethiopia. Ethiop J Health Sci.

[57] Imoudu IA, Zirami MG, Zamo AB, Idiodemise IE, Mahmoud LM, Sambo H (2020). Pattern of bacterial colonization of health care personnel at a reference hospital in North-eastern Nigeria. Highland Med Res J.

[58] Jamalludeen NM. Nasal carriage of *Staphylococcus aureus* in Healthy Children and its possible bacteriophage isolates in Basrah, Iraq. Biomed Pharmacol J. 2021;14(1).

[59] Gonzalbo FD, Rico VH, Falomir LMP, Gonzalbo FD, Rico VH, Falomir LMP (2019). Commensal Staphylococcus isolates from the nasal cavity of community older adults in Valencia (Spain) and their resistance to methicillin and other antibiotics. Eur J Health Res.

[60] Kapali S, Pokhrel A, Bastola A, Tuladhar R, Joshi DR (2022). Methicillin-resistant *Staphylococcus aureus* Nasal colonization in people living with HIV and Healthy people in Kathmandu, Nepal. Future Sci OA.

[61] Sakr A, Brégeon F, Mège JL, Rolain JM (2018). *Staphylococcus aureus* nasal colonization: an update on mechanisms, epidemiology, risk factors, and subsequent infections. Front Microbiol.

